# Effects of *Magnolia officinalis* extract on the growth performance and immune function of weaned piglets

**DOI:** 10.1186/s40813-025-00430-z

**Published:** 2025-04-03

**Authors:** Chen Zhang, Bifan Liu, Zhijuan Cui, Kunfu Wu, Haibo Huang, Yongliang Wang, Xiaokang Ma, Bi’e Tan

**Affiliations:** 1 Key Laboratory for Quality Regulation of Livestock and Poultry Products of Hunan Province, College of Animal Science and Technology, Hunan Agricultural University, Changsha, 410128 China; 2 Anhui Provincial Key Laboratory of Livestock and Poultry Product Safety, Institute of Animal Husbandry and Veterinary Medicine, Anhui Academy of Agricultural Sciences, Hefei, 230031 China; 3 Yuelushan Laboratory, Changsha, 410128 China; 4 Institute of Yunnan Circular Agricultural Industry, Pu’er, 665000 China

**Keywords:** *Magnolia officinalis* extract, Weaned piglet, Growth performance, Immunity function

## Abstract

**Background:**

*Magnolia officinalis* is a medicinal herb known for its pharmacological properties and as a potential natural feed additive. We aimed to assess the effects of dietary *Magnolia officinalis* extract (MOE) on the growth performance and immune function of piglets, and explored the potential of MOE as a natural alternative to antibiotics for piglet nutrition during weaning.

**Results:**

Compared with the basal diet group (CK), the MOE diet significantly increased average daily feed intake and reduced diarrhea incidence and serum interleukin-6 (IL-6) levels. Compared with 0.1% MOE group, the 0.05% MOE group had lower diarrhea rates, eosinophils (EOS) count, EOS’ percentage, and serum interleukin-4 levels. Compared with CK, 0.05% MOE supplementation in the diet could reduce the diarrhea incidence and the thymus index by elevating the levels of transforming growth factor-β (TGF-β) and interleukin-10 (IL-10) in the serum, jejunum, and ileum. Compared with the basal diet group, 0.05% MOE supplementation upregulated the mRNA expressions of *IL-10* and *TGF-β1* in the jejunum and ileum (*P* < 0.05) and those of *IL-10*, interleukin-1β (*IL-1β*), and interferon-γ (*IFN-γ*) in the thymus (*P* < 0.05). Moreover, 0.05% MOE increased the levels of butyric, isobutyric, isovaleric, and valeric acids in the colon.

**Conclusions:**

MOE supplementation could modulate the immune status of animals, lower production costs, and contribute to more sustainable and ethical pig farming practices by promoting healthier growth and reducing disease susceptibility. Our findings offer a sustainable solution to antibiotic use in animal farming, addressing concerns about antibiotic resistance and food safety.

**Graphical abstract:**

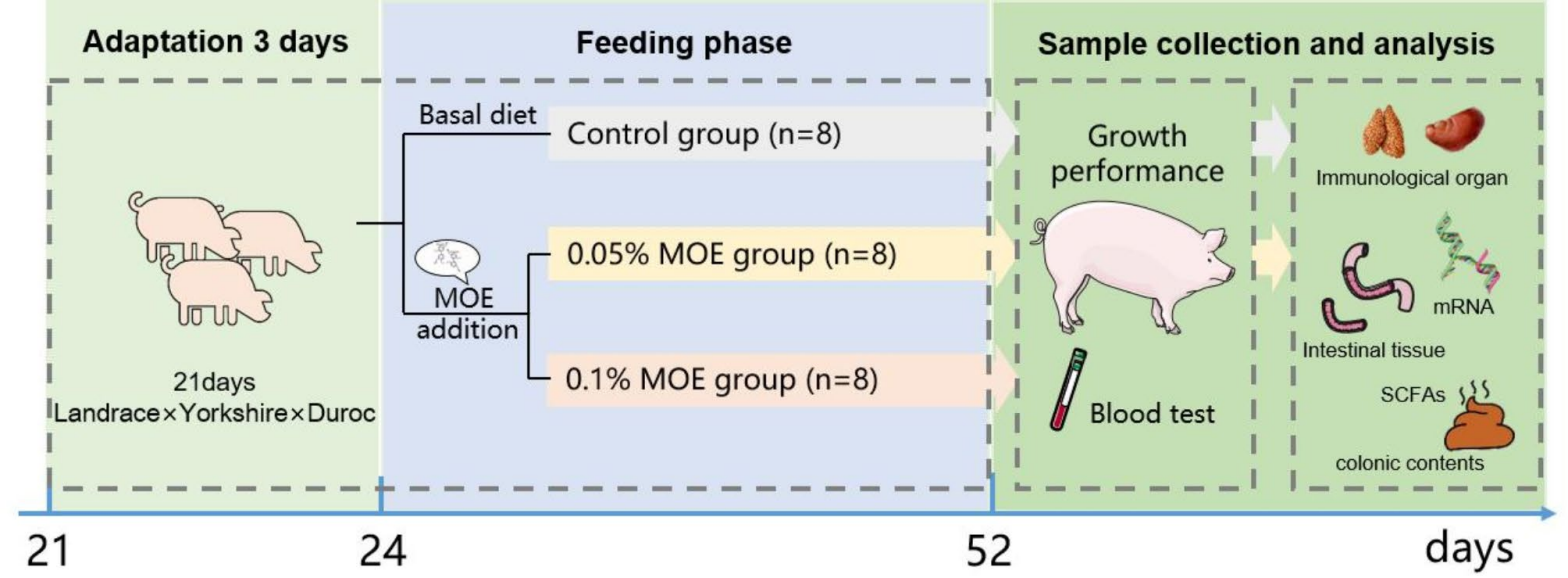

## Introduction

In modern intensive farming systems, early weaning is crucial to improve the efficiency of pig farming [1]. However, piglets have underdeveloped digestive and immune systems during the early weaning (weaning at 21 days), making them vulnerable to stressors like changes in feeding practices, environment, and nutrition [2, 3]. These stressors can alter the structure and function of the intestinal tract, reduce nutrient digestion and absorption, weaken the immune capacity, and increase susceptibility to diseases such as diarrhea [1, 4, 5]. Consequently, these factors reduced the productive performance of piglets and, in severe cases, even mortality. Since the 1950s, antibiotics have been widely used in animal agriculture to enhance animal growth and improve performance [6]. However, due to growing concerns about feed and food safety, issues related to the long-term use and misuse of antibiotics, including drug residues and antibiotic resistance, have been linked to human survival and health [7]. The European Union and China implemented a complete ban on antibiotics as growth promoters in livestock diets in January 2006 and July 2020, respectively [8, 9]. It is essential to identify alternatives or substitutes for antibiotics in pig feed to maintain profitability in the pig farming industry [10]. Specifically, there is a need to explore safe and effective nutritional strategies to enhance immune function, improve intestinal health, reduce the occurrence of weaning stress in piglets, alleviate the negative effects associated with weaning, and enhance the overall growth performance of piglets [11].

**Fig. 1:**
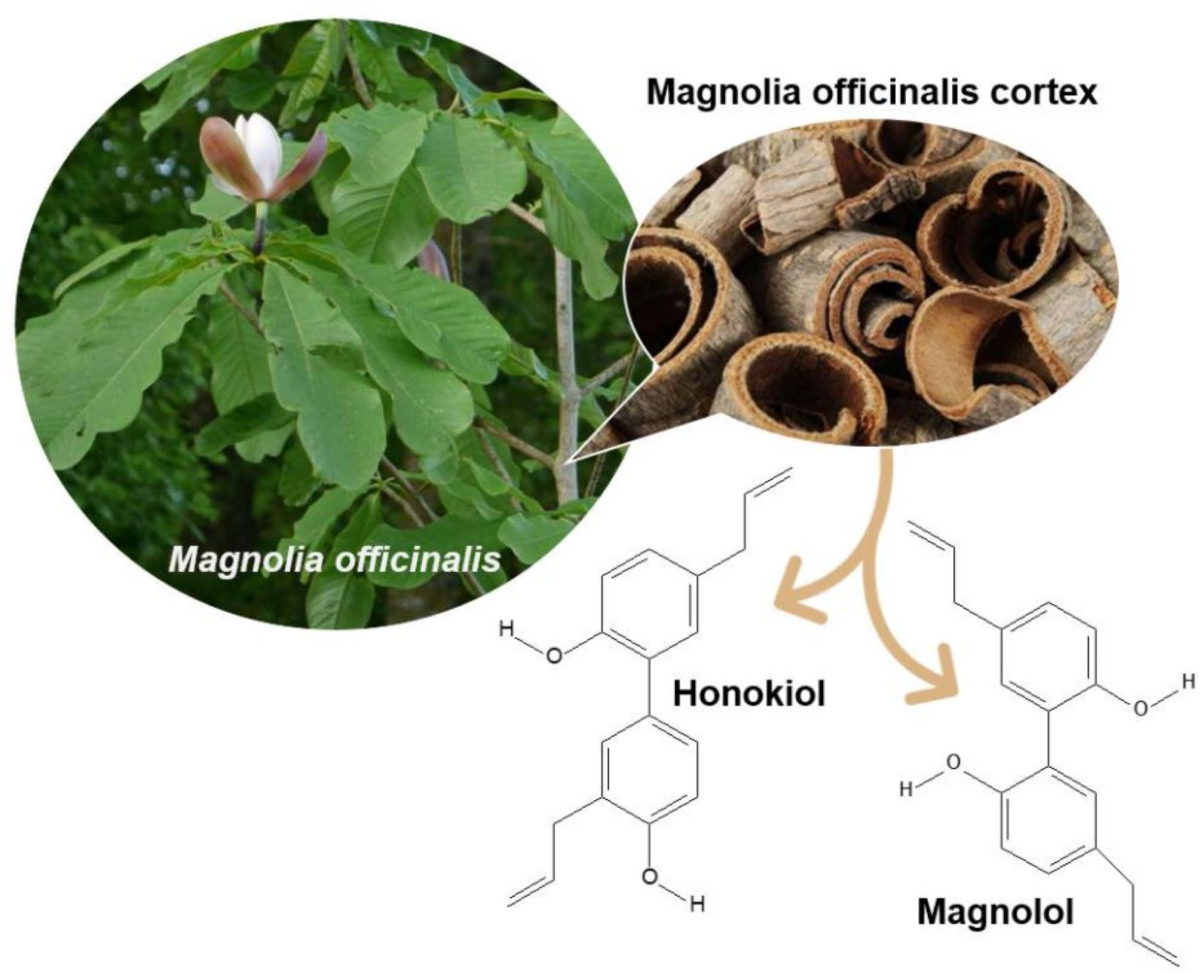
*Magnolia officinalis* and *Magnolia officinalis* (cortex) extract

Numerous studies have confirmed that plant extracts can promote animal growth, enhance immune function, and improve intestinal health. These extracts can be utilized as pure natural feed additives to alleviate weaning stress in piglets [12–15]. *Magnolia officinalis* cortex (the dried bark of *Magnolia officinalis*), commonly known as “Houpo,” has been used as a safe and reliable medicinal herb in Asian countries for thousands of years, including China, Japan, and Korea [16]. The main components of *Magnolia officinalis* (cortex) extract (MOE) are magnolol and honokiol (Fig. 1), which have several pharmacological functions [17, 18]. Total phenols of *Magnolia officinalis*, extracted from the bark, possess beneficial functional properties, including anti-inflammatory, analgesic, antimicrobial, anti-tumor, cardiovascular protection, anti-ulcer, anti-coagulation, and antioxidant activities [18–20]. This extract is primarily used in the pharmaceutical and health supplement industries. As a natural feed additive, MOE positively affects both animal health and product quality. MOE supplementation in finishing pigs delays lipid oxidation of raw meat and stimulates the Nrf2 pathway in liver cells to prevent oxidative stress [21, 22]. Dietary inclusion of MOE improves the growth performance and transcription of antioxidant enzymes in chickens [23]. MOE supplementation can alter the levels of certain metabolites absorbed in the chicken intestine [24].

Therefore, we hypothesized that MOE supplementation could promote the growth performance and immune function of weaned piglets. The aim of this study was to investigate the effects of 0.1% and 0.05% MOE supplementation on growth performance, diarrhea incidence, serum biochemical markers, and the levels of serum immunoglobulin and cytokines in weaned piglets and determine the appropriate MOE dosage. The effects of MOE supplementation on intestinal morphology, inflammatory factors, gene expression related to immune factors, and colonic short-chain fatty acids (SCFAs) were explored.

## Materials and methods

### Animals, management, and experimental design

Forty-eight barrow piglets ([Landrace × Yorkshire] × Duroc; 6.3 ± 0.2 kg) were weaned at 21 days and comprised a single weaning batch with similar genetic backgrounds. After three days of adaptation to the experimental environment, the piglets were randomly divided into three homogenous groups characterized by similar starting body weight, with eight replicates in each group and two piglets per replicate: control (basal diet), 0.05% MOE (basal diet supplemented with 0.05% MOE), and 0.1% MOE (basal diet supplemented with 0.1% MOE). The MOE product used in the experiment was provided by Hunan Health-Guard Bio-Tech Inc. (Yongzhou, China), and the dosages were selected based on the manufacturer’s recommended concentrations of either 0.05% or 0.1%. And *Magnolia officinalis* total phenols constituted 18.66% (magnolol, 8.06%, and honokiol, 10.6%, as analyzed by high performance liquid chromatography-mass spectrometry [25]). The nutritional level of the basic diet (Table 1) met the NRC [26] specifed nutritional needs for weaned piglets. All piglets were vaccinated following the farm’s regulations and were raised on plastic-slatted floors with free access to feed and water. Specifically, the Classical Swine Fever Vaccine, Live (China Pharmaceutical Group Animal Health Co., Ltd., Wuhan, China) was diluted with sterilized saline as per the instructions and administered via intramuscular or subcutaneous injection. The humidity and temperature in the room were automatically maintained at 70 ± 5% and the starting temperature was set at 27 °C, and it was gradually reduced by 1 °C each week, reaching a ending temperature of 23 °C. The animals were allowed to adapt to the new environment for three days before the beginning of the study, and the experiment lasted 28 days.

**Table 1:** Composition and nutrient levels in the basal diet (air-dried)

Ingredients	Content %
Extruded corn	22.00
Corn	40
Soybean meal (43% crude protein)	20.50
Whey powder (3% crude protein)	5.00
Fish meal (63% crude protein)	4.00
Glucose	2.00
Soybean oil	1.50
Limestone	1.33
Calcium hydrogen phosphate	0.88
Premix ^1^	1.00
Choline	0.10
Antioxidant	0.05
Citric acid	0.50
NaCl	0.10
98% L-lysine	0.53
DL-Methionine	0.27
L-Threonine	0.19
L-Tryptophan	0.05
Total	100.00
Ash^2^	5.62
Crude protein^2^	18.34
Ether extract^2^	3.27
Acid detergent fiber^2^	4.02
Neutral detergent fiber^2^	12.38
Ca^2^	0.71
SID P^2^	0.68
SID lysine ^2^	1.31
SID threonine ^2^	0.76
SID trptophan ^2^	0.25
Digestible energy (MJ/kg) ^3^	14.30

SID: standardized ileal digestibility

^1^ The premix provided the following per kilogram of the diet: VA 6450 IU, VD_3_ 2250 IU, VE 25 IU, VK 3 mg, VB_1_ 1.8 mg, VB_2_ 8 mg, VB_12_ 0.026 mg, folic acid 0.9 mg, biotin 4.5 mg, niacin 24 mg, pantothenic acid 20 mg, Zn 80 mg, Fe 150 mg, Cu 10 mg, Mn 4 mg, I 0.6 mg, Se 0.5 mg, Co 0.8 mg.

^2^ Nutrient levels were analyzed.

^3^ Nutrient levels were calculated.

### Sample collection

On the 28th day of the study, one piglet closest to the average body weight (BW) within each treatment from each replicate was chosen. Blood from the precaval vein was drawn to obtain the serum, which was stored at -80 °C for detecting biochemical indicators and immune factor levels. The optimal dosage (0.05% or 0.1%) was determined based on the improvement effect of MOE on piglet growth performance, serum biochemicals, and humoral immunity, followed by further assessment. Piglets from the control group and the optimal MOE dosage group were selected. One piglet closest to the average body weight (BW) within each treatment from each replicate was chosen, anesthetized by electrical stunning, and euthanized by exsanguination. The spleen and thymus were collected and weighed. A portion of the thymus was rapidly frozen in liquid nitrogen and stored at -80 °C to analyze immune factor gene expression. The colon contents were collected and stored at -80 °C to analyze SCFA concentrations. The middle parts of the jejunum and ileum segments were sampled and stored at -80 °C to analyze immune factor gene expression. The remaining samples were fixed in a 4% formaldehyde solution and stored for morphological analysis.

### Growth performance and diarrhea

Piglets were weighed at the beginning and end of the experiment after a 12-hour fast to calculate the average daily gain (ADG). Feed intake was recorded daily to calculate the average daily feed intake (ADFI) and the ratio of feed consumption to weight gain (feed/gain, F/G) [27]. Throughout the trial, the shape of the feces and the degree of fecal contamination surrounding the piglet’s anus were inspected at 09:00 and 16:00 daily, and scores were assigned to measure the occurrence and severity of diarrhea.

Fecal scoring was graded as follows: 0, normal; 1, soft stool; 2, mild diarrhea; 3, severe diarrhea. Fecal scores greater than 1 indicated diarrheal occurrence [28], and the diarrhea incidence was calculated using formula (1).

1 $$\:\text{D}\text{i}\text{a}\text{r}\text{r}\text{h}\text{e}\text{a}\:\text{i}\text{n}\text{c}\text{i}\text{d}\text{e}\text{n}\text{c}\text{e}\:\left(\text{\%}\right)=\sum\:\frac{A\times\:d}{T\times\:D}\times\:100$$

where *A* = the number of piglets with diarrhea per pen; *d* = days of diarrhea; *T* = total number of piglets; *D* = number of experimental days.

### Serum biochemistry and Immunologic factors

The levels of total protein (TP), albumin (ALB), alanine transaminase (ALT), aspartate aminotransferase (AST), lactic dehydrogenase (LDH), blood urea nitrogen (BUN), triglyceride (TG), cholesterol (CHOL), high-density lipoprotein (HDL), and low-density lipoprotein (LDL) in the serum of piglets were measured using an automatic biochemical analyzer (OLYMPUS AU400, Japan) following the instructions provided in the corresponding commercial assay kit (Shanghai Kehua Bio-Engineering Co., Ltd.). The ratio of AST to ALT was calculated. Peripheral blood cells, including white blood cells (WBC), neutrophils (NEU), lymphocytes (LYM), monocytes (MON), eosinophils (EOS), and basophilic granulocytes (BAS), were analyzed using an automatic hematology analyzer (HL-2400 Plus, Biotech, China) following the reagent-specific protocol (Nanchang Biotech A&C Biotechnical Co., Ltd.). The analysis included the absolute counts of these cell types and the percentages of NEU, LYM, MON, EOS, and BAS.

The serum levels of immunoglobulin M (IgM) and immunoglobulin G (IgG) were determined using an immunoassay analyzer (DxI 9000, Beckman, USA) following the instructions provided in the commercial assay kit (Shanghai Kehua Bio-Engineering Co., Ltd.). Concentrations of interleukin-1β (IL-1β), interleukin-4 (IL-4), interleukin-6 (IL-6), interleukin-10 (IL-10), and transforming growth factor-β (TGF-β) in the serum were determined using the corresponding enzyme-linked immunosorbent assay (ELISA) kits (CSB-E06782p, CSB-E06785p, CSB-E06786p, CSB-E06779p, CSB-E06843p, CUSABIO, https://www.cusabio.com/) following the manufacturer’s instructions.

### Immunological organ index, intestinal tissue morphology, and cytokine content in intestinal tissues

The immunological organ indices for the spleen and thymus were calculated using formula (2).

2 $$\:\text{O}\text{r}\text{g}\text{a}\text{n}\:\text{I}\text{n}\text{d}\text{e}\text{x}\:(\text{g}/\text{k}\text{g})=\frac{\text{O}\text{r}\text{g}\text{a}\text{n}\:\text{W}\text{e}\text{i}\text{g}\text{h}\text{t}\:\left(\text{g}\right)}{\text{B}\text{o}\text{d}\text{y}\:\text{W}\text{e}\text{i}\text{g}\text{h}\text{t}\:\left(\text{k}\text{g}\right)}$$

Jejunal and ileum samples were stained with hematoxylin and eosin (HE) or Alcian blue-periodic acid-Schiff (AB-PAS). Tissue slices were scanned using a PANNORAMIC panoramic slide scanner and Image-Pro Plus 6.0 was used for image analyses.

Ten well-oriented intact villi were selected from each section in each piglet, and the villous height and crypt depth (VH, CD; µm) were measured. Goblet cells and lymphocytes in the 10 villi were counted, and the mean of the 10 values of each sample was calculated. Concentrations of secretory immunoglobulin A (sIgA), IL-1β, IL-4, IL-6, IL-10, and TGF-β in the jejunal and ileal tissues were determined using the corresponding ELISA kits (CSB-E12063p, CSB-E06782p, CSB-E06785p, CSB-E06786p, CSB-E06779p, CSB-E06843p, CUSABIO, https://www.cusabio.com/) following the manufacturer’s instructions. Briefly, tissue samples were washed with pre-cooled physiological saline, and 500 µL of sterile PBS buffer (pH 7.2) was added. The samples were homogenized and crushed at 4 °C followed by centrifugation at 4 °C at 5000 × g for 10 min. The supernatant was collected for subsequent analysis.

### Composition of SCFAs in the colon

SCFA concentrations in the colon were determined following the procedure described by Wu et al. [29]. A fresh sample of the colon (1 g) was weighed and homogenized in 5 mL of distilled water, mixed thoroughly, and centrifuged at 4 °C at 10,000× g for 10 min to obtain supernatants. The obtained supernatants were diluted twice in ultra-pure water and centrifuged again at 4 °C at 10,000× g for 15 min. The supernatant was aspirated, mixed with 25% metaphosphoric acid at a volume ratio of 9:1, and allowed to stand at room temperature (27 ± 1 °C) for 3 to 4 h. Subsequently, the supernatant was centrifuged at 4 °C, 10,000 × g for 10 min, and filtered through a 0.22-µm membrane into a sample bottle. The sample was subjected to GC analysis (Agilent 6890, Palo Alto, CA).

### Expressions of immune genes in the jejunum, ileum, and thymus

The expressions of immune-related genes, including *IL-1β*, *IL-4*, *IL-10*, transforming growth factor-β1 (*TGF-β1*), tumor necrosis factor-α (*TNF-α*), and interferon-γ (*IFN-γ*) in the jejunum, ileum, and thymus were detected by real-time fluorescent quantitative PCR analysis. Total RNA was extracted from tissue samples using the TRIzol reagent (Invitrogen, USA), and cDNA was synthesized from the extracted total RNA using a reverse transcription kit (Takara Inc., Japan) following the manufacturer’s instructions. Real-time PCR amplification was performed using the TB Green^®^ *Premix EX Taq*™ II kit (Takara Inc., Japan) on a Lightcycler 480 (Roche, USA). Kit-specific instructions were followed; specifically, a 10-µL volume containing 5 µL of TB Green Premix EX Taq II (2X), 0.5 µL of forward and reverse primers (10 µM), and 4 µL of cDNA template was used for real-time PCR analysis. The thermal cycling conditions were as follows: pre-denaturation (30 s at 95 °C), amplification (at 95 °C for 5 s and 60 °C for 30 s, 40 cycles), melting curve construction (60 to 95 °C with a heating rate of 0.2 °C/s), and fluorescence measurements. *β-actin* was the internal reference, and the relative expressions of target genes were calculated using the 2^−ΔΔCt^ method. Primer sequences used in this study are provided in Table 2.

**Table 2:** Primer sequence used in PCR analysis

Gene	Sequence 5’-3’	Size, bp	Accession No.
*β-Actin*	F: CTGCGGCATCCACGAAACT R: AGGGCCGTGATCTCCTTCTG	147	XM_003124280.5
*IL-1β*	F: CAGCCAGTCTTCATTGTTCAGG R: GTTTTGGGTGCAGCACTTCAT	150	NM_214055.1
*IL-4*	F: CCCGAGTGTCAAGTGGCTTA R: TGATGATGCCGAAATAGCAG	122	NM_214340.1
*IL-10*	F: GGGCTATTTGTCCTGACTGC R: GGGCTCCCTAGTTTCTCTTCC	105	NM_214041.1
*TGF-β1*	F: AAGCGGCAACCAAATCTATG R: CCCGAGAGAGCAATACAGGT	113	NM_214015.2
*TNF-α*	F: GGCCCAAGGACTCAGATCAT R: CTGTCCCTCGGCTTTGACAT	82	NM_214022.1
*IFN-γ*	F: TTCAGCTTTGCGTGACTTTG R: GGTCCACCATTAGGTACATCTG	121	NM_213948.1

IL-1β, Interleukin-1β; IL-4, Interleukin-4; IL-10, Interleukin-10; TGF-β1, Transforming growth factor-β1; TNF-α, Tumor necrosis factor-α; IFN-γ, Interferon-γ.

### Statistical analysis

The replicate was used as a statistical unit, and all data were tested for normality and homogeneity of variance using the Kolmogorov–Smirnov and Levene tests (with the significance level set at 5%) in SPSS 23.0 (SPSS, Inc., Chicago, USA). Then with experimental diet (Control, 0.05% MOE and 0.1% MOE) as a fixed effect, experimental data on growth performance, serum biochemistry, peripheral blood cells, and the levels of serum immunoglobulin and cytokines were analyzed using a one-way ANOVA and Duncan’s post hoc multiple comparison tests, and diarrhea incidence was analyzed using Kruskal-Wallis test; With experimental data on intestinal morphology, immune organ indexes, tissue cytokines, expression of immune-related genes, and SCFAs as random factors, unpaired t-tests were employed to compare the control and 0.05% MOE groups. *P* < 0.05 indicated a statistically significant difference, and 0.05 ≤ *P* < 0.10 indicated a significant trend.

## Results

### Effects of the MOE diet on growth performance and diarrhea incidence in weaned piglets

The effects of the MOE diet on growth performance and diarrhea incidence in weaned piglets are presented in Table 3. Compared with the control group, the MOE diet (0.05% and 0.1% MOE diets) increased ADFI (*P* = 0.007) and decreased diarrheal incidence in weaned piglets (*P* < 0.01), and the diarrhea incidence of 0.05% group was the lowest. There were no significant differences in the BW and F/G among the groups (*P* > 0.05) but 0.05% and 0.1% MOE diets showed a trend toward improved ADG of piglets (*P* = 0.067). And compared with 0.1% MOE, Supplementation with 0.05% MOE in the diet decreased diarrheal incidence in weaned piglets (*P* < 0.01). Therefore, the 0.05% MOE and control groups were selected for slaughter.

**Table 3:** Effects of *Magnolia officinalis* extract (MOE) supplementation on growth performance of weaned piglets

Items	Control	MOE		SEM	*P*-value
		0.05%	0.1%		
Initial BW (kg)	7.38	7.39	7.33	0.39	0.986
Final BW (kg)	15.34	16.13	15.82	0.38	0.360
ADG (g/d)	284.49	312.10	303.13	7.23	0.067
ADFI (g/d)	582.15^b^	659.42^a^	668.28^a^	20.53	0.007
F/G	2.05	2.12	2.22	0.11	0.414
Diarrhea incidence (%)	32.42^a^	24.53^c^	28.60^b^	1.01	< 0.001

BW, bodyweight; ADG, average daily gain; ADFI, average daily feed intake; F/G, feed/gain. In the same row, values with no letter or the same letter superscripts indicate no significant difference (*P* > 0.05). In contrast, values with different letter superscripts imply a significant difference (*P* < 0.05); 0.05 ≤ *P* < 0.10 indicates a significant trend. Data are presented as mean ± SEM (*n* = 8)

### Effects of dietary MOE on serum biochemical indicators of weaned piglets

As shown in Table 4, there was no difference in the serum levels of TP, ALB, ALT, AST, LDH, BUN, TG, CHOL, LDL, and HDL among the three groups (*P* > 0.05). However, dietary supplementation with MOE showed a trend toward decreased the ratio of AST to ALT (*P* = 0.052) compared with the control group.

**Table 4:** Effects of *Magnolia officinalis* extract (MOE) on serum biochemical parameters in weaned piglets

Items	Control	MOE		SEM	*P*-value
		0.05%	0.1%		
TP (g/L)	46.41	47.28	47.44	1.82	0.832
ALB (g/L)	31.20	32.60	29.31	2.5	0.445
ALT (U/L)	46.57	47.11	48.00	2.71	0.868
AST (U/L)	40.50	39.86	41.38	4.15	0.935
AST / ALT	0.98	0.82	0.83	0.07	0.052
LDH (U/L)	476.25	523.00	526.63	35.77	0.311
BUN (mmol/L)	1.34	1.70	1.26	0.27	0.342
TG (mmol/L)	0.43	0.46	0.44	0.04	0.779
CHOL (mmol/L)	2.22	2.13	2.37	0.26	0.651
LDL (mmol/L)	1.21	1.21	1.34	0.19	0.741
HDL (mmol/L)	0.82	0.72	0.83	0.12	0.638

TP, total protein; ALB, albumin; ALT, alanine transaminase; AST, aspartate aminotransferase; AST / ALT, the ratio of AST to ALT; LDH, lactic dehydrogenase; BUN, blood urea nitrogen; TG, triglyceride; CHOL, cholesterol; HDL, high-density lipoprotein; LDL, low-density lipoprotein. In the same row, values with no letter or the same letter superscripts indicate no significant difference (*P* > 0.05). In contrast, values with different letter superscripts represent a significant difference (*P* < 0.05); 0.05 ≤ *P* < 0.10 implies a significant trend. Data are presented as mean ± SEM (*n* = 8).

### Effects of dietary MOE on the humoral immunity of weaned piglets

As shown in Table 5, compared with the control group, dietary supplementation with 0.05% MOE significantly decreased the EOS count in the peripheral blood of weaned piglets (*P* < 0.05), while 0.1% MOE significantly increased the EOS count and EOS’ percentage to WBC (*P* < 0.05), the EOS count and EOS’ percentage to WBC were the lowest (*P* < 0.05) in the 0.05% MOE group. As shown in Table 6, compared with the control group, dietary supplementation with MOE decreased IL-6 levels (*P* < 0.05), which was the lowest in the 0.05% MOE group. Dietary supplementation with 0.05% MOE increased the serum TGF-β levels. Dietary supplementation with 0.05% MOE decreased the serum IL-4 levels (*P* < 0.05), and increased those of IgG and IL-10 (*P* < 0.05) compared with the 0.1% MOE group.

**Table 5:** Effects of *Magnolia officinalis* extract (MOE) on the differential blood count of weaned piglets

Items		Control	MOE		SEM	*P*-value
			0.05%	0.1%		
Count (10^9^/L)	WBC	20.11	20.44	23.13	1.03	0.431
	NEU	4.80	4.67	6.09	0.57	0.551
	LYM	14.18	14.53	15.12	0.63	0.831
	MON	0.95	1.07	1.51	0.14	0.216
	EOS	0.16^b^	0.12^c^	0.35^a^	0.35	0.011
	BAS	0.03	0.05	0.05	0.01	0.126
Percentage (%)	NEU	23.94	21.33	25.45	1.89	0.539
	LYM	70.44	73.12	66.47	2.14	0.443
	MON	4.70	4.75	6.37	0.44	0.715
	EOS	0.77^b^	0.57^c^	1.50^a^	0.14	0.003
	BAS	0.14	0.23	0.21	0.02	0.558

WBC, white blood cell; NEU, neutrophil; LYM, lymphocyte; MON, monocyte; EOS, eosinophils; BAS, basophilic granulocyte. In the same row, values with no letter or the same letter superscripts indicate no significant difference (*P* > 0.05). In contrast, values with different letter superscripts imply a significant difference (*P* < 0.05); 0.05 ≤ *P* < 0.10 indicates a significant trend. Data are presented as mean ± SEM (*n* = 8).

**Table 6:** Effects of *Magnolia officinalis* extract (MOE) on serum Immunoglobulin and cytokine levels in weaned piglets

Items	Control	MOE		SEM	*P*-value
		0.05%	0.1%		
IgM (g/L)	0.70	0.66	0.65	0.09	0.858
IgG (g/L)	3.24^b^	4.35^a^	3.42^b^	0.29	0.002
IL-1β (pg/mL)	129.49	124.49	125.36	5.27	0.605
IL-4 (pg/mL)	11.13^b^	11.40^b^	12.77^a^	0.43	0.002
IL-6 (pg/mL)	191.23^a^	167.93^b^	171.24^b^	4.63	< 0.001
IL-10 (pg/mL)	27.45^b^	30.69^a^	26.24^b^	0.97	0.001
TGF-β (pg/mL)	154.05^b^	176.11^a^	165.03^ab^	5.63	0.003

IgM, immunoglobulin M; IgG, immunoglobulin G; IL-1β, interleukin-1β; IL-4, interleukin-4; IL-6, interleukin-6; IL-10, interleukin-10; TGF-β, transforming growth factor-β. In the same row, values with no letter or the same letter superscripts indicate no significant difference (*P* > 0.05). In contrast, values with different letter superscripts imply a significant difference (*P* < 0.05); 0.05 ≤ *P* < 0.10 indicates a significant trend. Data are presented as mean ± SEM (*n* = 8).

### Effects of dietary supplementation with 0.05% MOE on the intestinal morphology of weaned piglets

**Fig. 2:**
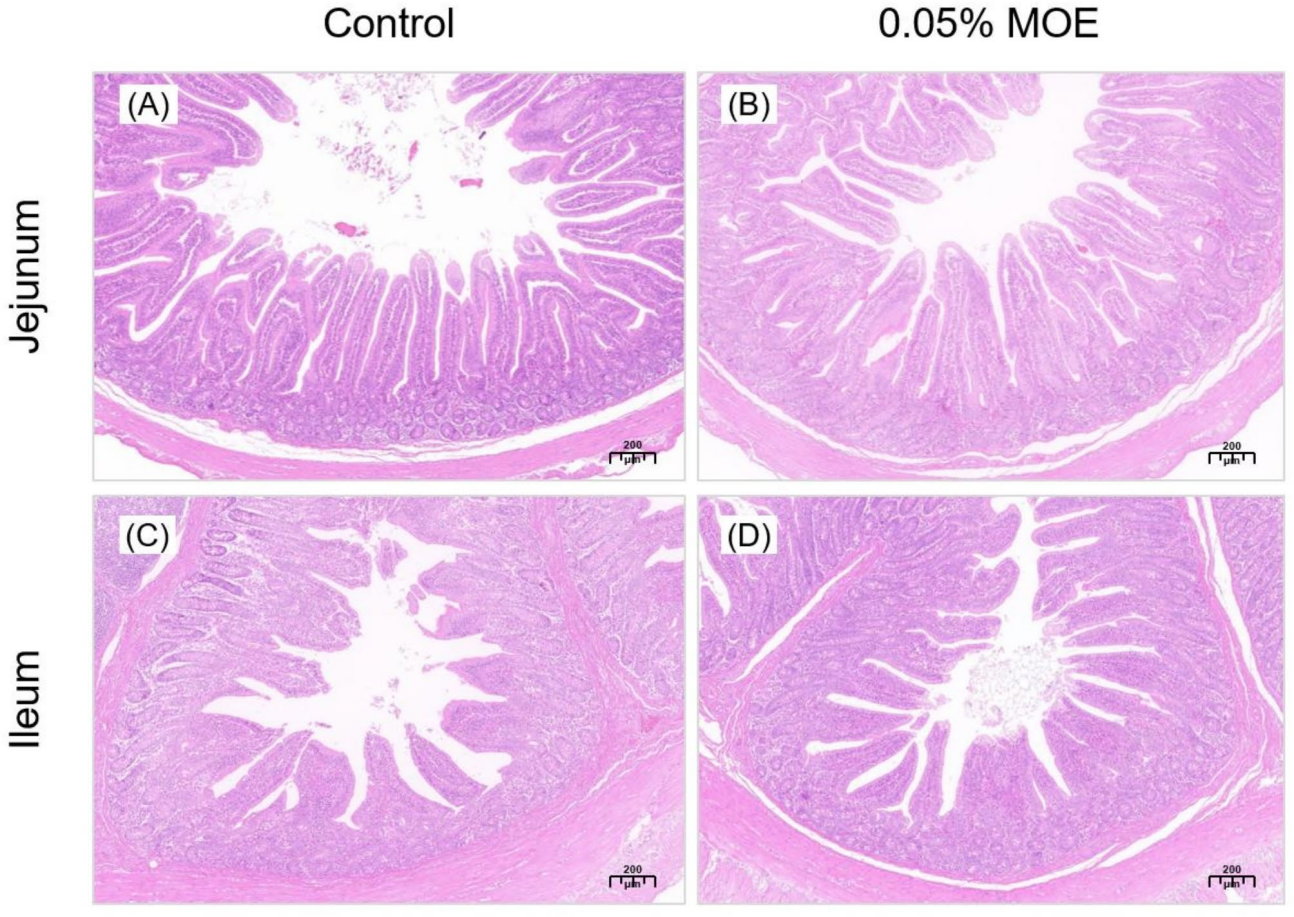
Effect of dietary *Magnolia officinalis* extract (MOE) on the intestinal morphology of the jejunum and ileum in weaned piglets. (**A**, **B**) Jejunal sections of piglets in the control and 0.05% MOE groups. (**C**, **D**) Ileum section of piglets in the control and 0.05% MOE groups

Based on the results of growth performance, serum biochemistry, and humoral immunity, the samples of piglets from the control and the 0.05% MOE groups were selected for further investigation. The morphology data of the jejunum and ileum, and the numbers of goblet cells and lymphocytes are presented in Table 7; Fig. 2. As shown in Fig. 2, there were no obvious pathological changes between the two groups in intestinal morphology. The VH of jejunum was higher in the 0.05% MOE group compared to the control group (*P* < 0.05) and showed a trend toward increased the goblet cell number (*P* = 0.065) in jejunum (Table 7). Furthermore, 0.05% MOE increased the number of lymphocytes (*P* < 0.05) in ileum. These results suggest that dietary 0.05% MOE supplementation is not harmful to the intestinal health of piglets and can improve the morphological structure of the small intestine to some extent. In the ileum, this improvement may be attributed to an increase in the number of lymphocytes.

**Table 7:** Effects of 0.05% *Magnolia officinalis* extract (MOE) on the intestinal morphology of weaned piglets

Items		Control	0.05% MOE	SEM	*P*-value
Jejunum	VH (µm)	378.51^b^	414.24^a^	14.54	0.028
	CD (µm)	167.8	184.00	18.09	0.386
	VH: CD	2.39	2.31	0.28	0.792
	Goblet cell number	8.20	9.20	0.51	0.065
	Lymphocyte number	93.27	114.51	16.16	0.210
Ileum	VH (µm)	349.82	376.62	30.66	0.458
	CD (µm)	163.14	160.89	14.34	0.878
	VH: CD	2.17	2.38	0.26	0.440
	Goblet cell number	18.50	18.30	1.14	0.863
	Lymphocyte number	36.94^b^	57.25^a^	8.36	0.029

VH, villous height; CD, crypt depth; VH: CD, villus height to crypt depth ratio. In the same row, values with no letter or the same letter superscripts indicate no significant difference (*P* > 0.05). In contrast, values with different letter superscripts imply a significant difference (*P* < 0.05); 0.05 ≤ *P* < 0.10 indicates a significant trend. Data are presented as mean ± SEM (*n* = 8).

### Effects of dietary supplementation with 0.05% MOE on the intestinal immune function and immune organs of weaned piglets

**Fig. 3:**
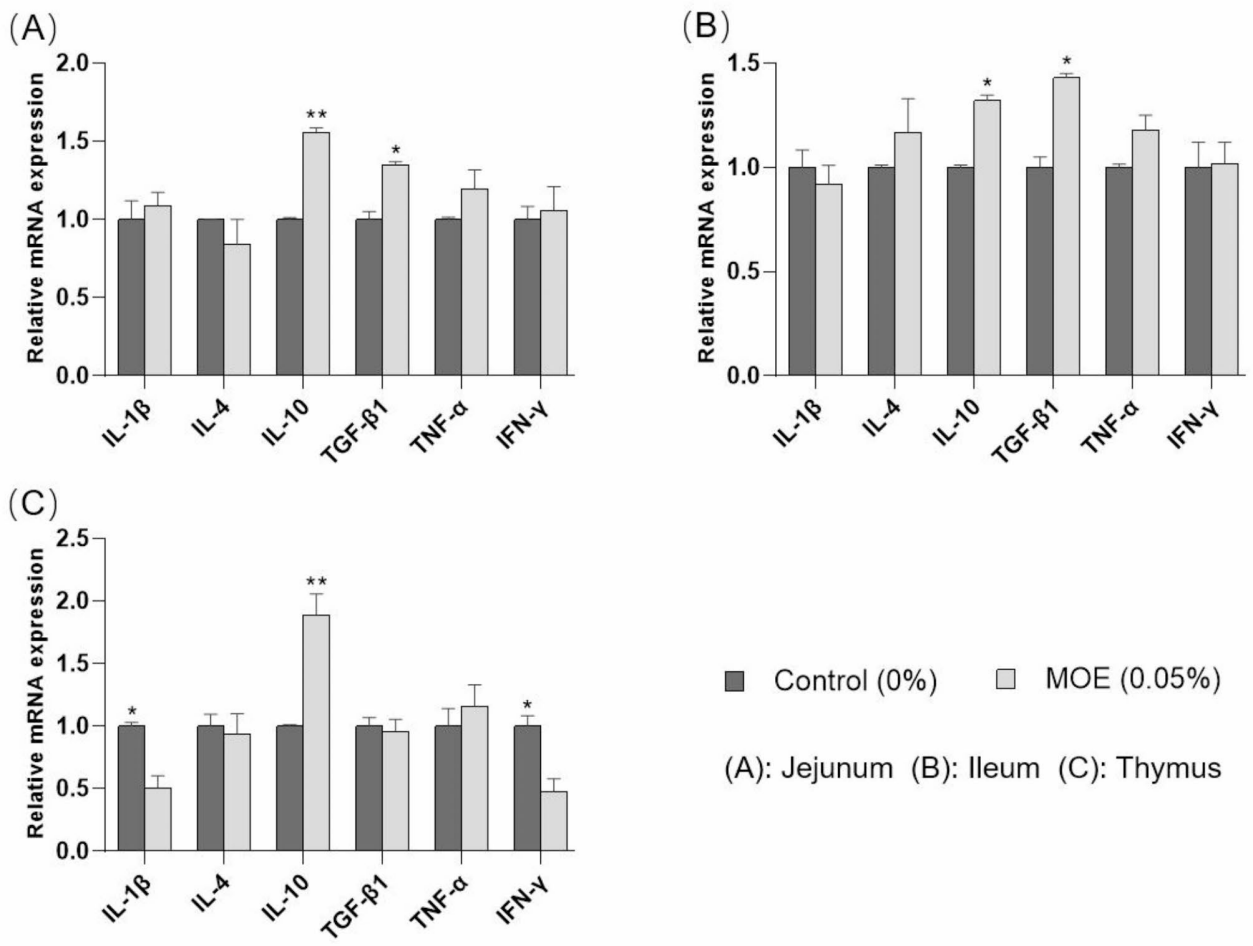
mRNA expressions of immune-related genes in weaned piglets. IL-1β, interleukin-1β; IL-4, interleukin-4; IL-10, interleukin-10; TGF-β1, transforming growth factor-β1; TNF-α, tumor necrosis factor-α; IFN-γ, interferon-γ; *: *P* < 0.05; **: *P* < 0.01

Immune organ indices and cytokine levels are presented in Tables 8 and 9. Dietary supplementation with 0.05% MOE significantly increased the thymus index (*P* < 0.05), compared with the control group, but there were no significant differences between the two groups in terms of the spleen index (*P* > 0.05) (Table 8). IL-10 and TGF-β levels in the jejunum and those of sIgA and IL-10 in the ileum were higher in the 0.05% MOE group than those in the control group (*P* < 0.05) (Table 9). In addition, 0.05% MOE group notably decreased the pro-inflammatory levels of IL-1β and IL-6 in the jejunum. Therefore, we further detected the mRNA expressions of immune-related genes in the jejunum, ileum, and thymus. As shown in Fig. 3; Table 10, dietary supplementation with 0.05% MOE increased the mRNA expressions of IL-10 and TGF-β1 in the jejunum and ileum (*P* < 0.05) (Fig. 3A, B), and that of the IL-10 mRNA in the thymus (*P* < 0.05) (Fig. 3C). Supplementation with 0.05% MOE showed a trend toward decreased IL-4 mRNA expression in the jejunum and ileum (0.05 < *P* < 0.10) and significantly decreased the mRNA expressions of IL-1β and IFN-γ in the thymus (*P* < 0.05) (Table 10).

**Table 8:** Effects of 0.05% *Magnolia officinalis* extract (MOE) on the immune organ index of weaned piglets

Items (g/kg)	Control	0.05% MOE	SEM	*P*-value
Spleen index	2.12	2.45	0.21	0.134
Thymus index	0.71^b^	0.94^a^	0.08	0.016

In the same row, values with no letter or the same letter superscripts indicate no significant difference (*P* > 0.05). In contrast, values with different letter superscripts imply a significant difference (*P* < 0.05); 0.05 ≤ *P* < 0.10 indicates a significant trend. Data are presented as mean ± SEM (*n* = 8).

**Table 9:** Effects of 0.05% *Magnolia officinalis* extract (MOE) on the tissue cytokines of jejunum and ileum in weaned piglets

Items		Control	0.05% MOE	SEM	*P*-value
Jejunum	sIgA (µg/mL)	137.71	133.99	18.80	0.846
	IL-1β (pg/mL)	110.22^a^	84.80^b^	4.10	< 0.001
	IL-4 (pg/mL)	11.23	10.90	1.60	0.839
	IL-6 (pg/mL)	148.30^a^	114.57^b^	3.92	< 0.001
	IL-10 (pg/mL)	20.88^b^	24.97^a^	1.47	0.015
	TGF-β (pg/mL)	293.18^b^	414.95^a^	51.11	0.032
Ileum	sIgA (µg/mL)	134.09^b^	162.21^a^	10.33	0.016
	IL-1β (pg/mL)	110.43	121.95	9.26	0.234
	IL-4 (pg/mL)	11.84	12.68	0.90	0.371
	IL-6 (pg/mL)	147.97	160.89	12.71	0.326
	IL-10 (pg/mL)	24.18^b^	29.43^a^	1.69	0.008
	TGF-β (pg/mL)	270.82	251.96	15.05	0.231

In the same row, values with no letter or the same letter superscripts indicate no significant difference (*P* > 0.05). In contrast, values with different letter superscripts imply a significant difference (*P* < 0.05); 0.05 ≤ *P* < 0.10 indicates a significant trend. Data are presented as mean ± SEM (*n* = 8).

**Table 10:** mRNA expressions of immune-related genes in weaned piglets

Items		Control	0.05% MOE	SEM	*P*-value
Jejunum	IL-1β	1.00	1.09	0.05	0.116
	IL-4	1.00	0.84	0.09	0.087
	IL-10	1.00^b^	1.56^a^	0.08	< 0.001
	TGF-β1	1.00^b^	1.21^a^	0.08	0.027
	TNF-α	1.00	1.19	0.11	0.102
	IFN-γ	1.00	1.24	0.13	0.100
Ileum	IL-1β	1.00	0.92	0.07	0.283
	IL-4	1.00	1.17	0.08	0.051
	IL-10	1.00^b^	1.34^a^	0.08	0.002
	TGF-β1	1.00^b^	1.42^a^	0.08	< 0.001
	TNF-α	1.00	1.19	0.11	0.102
	IFN-γ	1.00	1.02	0.10	0.871
Thymus	IL-1β	1.00^a^	0.51^b^	0.08	< 0.001
	IL-4	1.00	0.83	0.10	0.105
	IL-10	1.00^b^	1.89^a^	0.11	< 0.001
	TGF-β1	1.00	0.96	0.06	0.463
	TNF-α	1.00	1.16	0.10	0.121
	IFN-γ	1.00^a^	0.48^b^	0.08	< 0.001

In the same row, values with no letter or the same letter superscripts indicate no significant difference (*P* > 0.05). In contrast, values with different letter superscripts imply a significant difference (*P* < 0.05); 0.05 ≤ *P* < 0.10 indicates a significant trend. Data are presented as mean ± SEM (*n* = 8).

### Composition of SCFAs in the colon

The composition of SCFAs in the colonic contents is shown in Table 11. Dietary supplementation with 0.05% MOE markedly increased the levels of Total SCFAs. At the same time, the content of isobutyric, isovaleric, and valeric acids in the colon were significantly higher than those in the control group (*P* < 0.05).

**Table 11:** Effects of MOE on short-chain fatty acid (SCFA) concentrations in the colonic contents of weaned piglets

Items (µg/g)	Control	0.05% MOE	SEM	*P*-value
Acetic acid	297.56	305.93	26.67	0.758
Propionic acid	363.74	397.97	41.45	0.423
Butyric acid	638.37	782.66	91.25	0.136
Isobutyric acid	69.35^b^	116.52^a^	10.32	< 0.001
Isovaleric acid	264.65^b^	352.04^a^	25.68	0.004
Valeric acid	187.33^b^	256.75^a^	21.27	0.006
Total SCFAs	1842.85^b^	2211.87^a^	146.27	0.024

In the same row, values with no letter or the same letter superscripts indicate no significant difference (*P* > 0.05). In contrast, values with different letter superscripts imply a significant difference (*P* < 0.05); 0.05 ≤ *P* < 0.10 indicates a significant trend. Data are presented as mean ± SEM (*n* = 8).

## Discussion

Chinese herbal medicine, as feed additives, promotes the growth of livestock and poultry, provides health care, prevents disease, and has non-toxic, harmless, non-residual, and non-resistant properties [30]. Therefore, they are considered a potential alternative to antibiotic additives, effectively mitigating the spread of antibiotic resistance (AMR) and the global health threat posed by AMR [31, 32]. Zhang et al. [33] reported significantly increased ADG and feed conversion ratio in broilers following dietary supplementation with a composite herbal medicine comprising *Magnolia officinalis* and hawthorn fruit. Qu et al. indicated that magnolol improved the average daily weight gain of weaned piglets [34]. Xia et al. [35] found that Cangpo oral liquid (CAL) contains 130 µg/mL of magnolol and 300 µg/mL of honokiol, and its oral administration inhibited small intestine peristalsis in piglets with diarrhea, resulting in an enhanced recovery rate in piglet diarrhea. Deng’s study demonstrated that MOE reduced diarrhea by decelerating the creeping speed of the small intestines and blocking the Ca^2+^ channel [36]. In this study, supplementation with 0.05% and 0.1% MOE increased piglet ADFI and reduced the diarrhea incidence in experimental piglets, consistent with previous results. Some studies have shown that MOE can inhibit pathogenic bacteria such as *Escherichia coli* and *Salmonella*, and it also has inhibitory effects on Gram-positive bacteria [37]. Supplementing with a high dose (0.1%) of MOE may inhibit intestinal pathogenic bacteria but also suppress lactic acid bacteria, leading to intestinal microbiota imbalance and inflammation [38]. This resulted in an increased EOS count, a higher percentage of EOS in WBC, and elevated serum IL-4 level in the 0.1% MOE group, which may also explain why the diarrhea incidence in the 0.1% MOE group was higher than in the 0.05% MOE group and not higher than the CK group.

Intestinal morphology and the integrity of the intestinal mucosa are important indicators of intestinal health and function [39]. Increased VH implies an increase in the digestibility of nutrients in the intestine [40]. Dietary supplementation with 0.01–0.03% MOE positively affects the intestinal mucosal structure of broilers [41]. Similarly, Lin’s study showed that meat ducks fed magnolol had intact intestinal mucosa and magnolol increased the VH in the duodenum and ileum of meat ducks [42]. In this study, dietary supplementation with 0.05% MOE increased the VH of the ileum, indicating that MOE promoted the growth and repair of intestinal epithelial cells (**IECs**). As a crucial component of the ileum immune system, ileum lymphocytes can recognize and eliminate invading pathogens and other foreign substances. We observed an increase in the number of lymphocytes in the ileum, indicating that a diet with 0.05% MOE improved the animals’ immune defense against pathogens and other external threats. MOE had no significant effects on VH and CD in the ileum. This may be due to the varying absorption capabilities of different segments of the intestine for MOE [43], and suggests that MOE may affect the intestinal mucosa differently at different locations. Therefore, future studies should focus on investigating these variations in different parts of the intestine to maximize the potential benefits of MOE.

*Magnolia officinalis* effectively ameliorated intestinal pathological damage and barrier gene expression [44]. Cheng et al. [45] discovered that magnolol enhanced the immune response and immune organ index in broilers, contributing to increased disease resistance. The addition of 200 mg/kg magnolol in the diet upregulated the relative expression of claudin proteins in the jejunum of laying hens, thereby maintaining the intestinal mucosal barrier function [46]. In a study on goldfish, Zhang et al. found that dietary supplementation with magnolol (3 g magnolol/kg commercial feed) elevated the expressions of anti-inflammatory cytokines (IL-10, TGF-β, and IL-4) in the liver [47]. As a free radical scavenger, magnolol decreased cerebral infarction volume and neuronal apoptosis in traumatic brain injury in rats by decreasing hydroxyl radical levels and upregulating TGF-β1 expression [48]. Moreover, MOE exerts anti-inflammatory effects by inhibiting the TLR/MAPK/NF-κB signaling pathway [49]. Magnolol also promotes the secretion of IL-10, which in turn reduces the production of pro-inflammatory cytokines such as IL-6, IL-8, and IL-1β [50]. In this study, dietary supplementation with MOE enhanced the immune function of weaned piglets and reduced the number of blood EOS and serum IL-6 levels. Piglets supplemented with 0.05% MOE showed significantly higher thymus organ index and levels of IgG, IL-10, and TGF-β in serum, expression of sIgA in the ileum, and the ileum lymphocyte number compared with the control group. Piglets in the 0.1% MOE groups showed significantly higher serum IL-4 levels than the control group. Supplementation with 0.05% MOE significantly increased the levels of IL-10 and TGF-β1 in the intestine and IL-10 mRNA expression in the thymus. Concurrently, it decreased the levels of IL-1β and IL-6, as well as IL-1β and IFN-γ mRNA expression in the thymus. These results of this study are consistent with previous findings, demonstrating MOE’s ability to regulate metabolism and improves immune function.

Furthermore, MOE could significantly increase the relative abundance of beneficial microorganisms [44], and improve the intestinal flora of mic, and maintain normal metabolic pathways of gut microbiota [51]. SCFAs derived from the fermentation of the gut microbiome crucially maintain intestinal homeostasis [52], they serve as an essential energy source for IECs and strengthen the gut barrier functions [53], and SCFAs exert anti-inflammatory effects in the intestinal mucosa by inhibiting histone deacetylases and activating the G-protein-coupled receptors of IECs and immune cells [54]. Oral administration of SCFAs as bacterial fermentation products promotes intestinal cell proliferation in both germ-free and specific pathogen-free mice [55]. Valeric acid can increase the density of glucagon-like peptide-2-producing enteroendocrine cells and reduce the incidence of necrotic enteritis [56]. Li et al. comfirmed that the valeric acid derived by gut commensal elevated the survival rate of irradiated mice, protected hematogenic organs, improved gastrointestinal tract function, and maintained intestinal epithelial integrity [57]. Mei et al. found that dietary supplemented 0.02% magnolol significantly increased the contents of acetate, propionate and SCFAs in the feces of weaned piglets [58], and we observed an increase in the levels of Total SCFAs, isobutyric acid, isovaleric acid, and valeric acid in the colon, when piglets supplemented with 0.05% MOE. Taken together, MOE can potentially enhance animal intestinal homeostasis by increasing the levels of SCFAs in the intestine.

## Conclusions

In this study, dietary supplementation with 0.05% and 0.1% MOE both significantly increased the ADFI, reduced the diarrhea incidence, and lowered serum IL-6 levels in weaned piglets, indicating a positive effect on gut health and inflammation. However, the effects were more pronounced with 0.05% MOE, particularly in improving growth performance, serum biochemistry, and humoral immune responses. The 0.05% MOE diet enhanced the expression of immune-related genes, including *IL-10* and *TGF-β1*, in the thymus and intestine, while also increasing the levels of SCFAs in the colon. These findings highlight that 0.05% MOE supplementation can reduce diarrhea incidence, enhance immune response, and improve gut health, suggesting its potential as an effective and valuable feed additive for weaned piglets.

## Data Availability

No datasets were generated or analysed during the current study.

## Abbreviations

AB-PAS: Alcian blue-periodic acid-Schiff
ADFI: Average daily feed intake
ADG: Average daily gain
ALB: Albumin
ALT: Alanine transaminase
AST: Aspartate aminotransferase
BAS: Basophilic granulocytes
BUN: Blood urea nitrogen
BW: Body weight
CD: Crypt depth
CHOL: Cholesterol
CK: Basal diet group
ELISA: Enzyme-linked immunosorbent assay
EOS: Eosinophils
F/G: Feed/gain
HDL: High-density lipoprotein
HE: Hematoxylin and eosin
IFN-γ: Interferon-γ
IgM: Immunoglobulin M
IgG: Immunoglobulin G
IL-1β: Interleukin-1β
IL-4: Interleukin-4
IL-6: Interleukin-6
IL-10: Interleukin-10
LDH: Lactic dehydrogenase
LDL: Low-density lipoprotein
LYM: Lymphocytes
MOE: Magnolia officinalis extract
MON: Monocytes
NEU: Neutrophils
SCFAs: Short-chain fatty acids
SID: Standardized ileal digestibility
sIgA: Secretory immunoglobulin A
TG: Triglyceride
TGF-β: Transforming growth factor-β
TNF-α: Tumor necrosis factor-α
TP: Total protein
VH: Villous height
WBC: White blood cells
